# Variation in antibody titers determined by Abbott and Roche Elecsys SARS-CoV-2 assays in vaccinated healthcare workers

**DOI:** 10.1016/j.heliyon.2023.e16547

**Published:** 2023-05-22

**Authors:** Miku Nakai, Daisuke Yokoyama, Tomoaki Sato, Ryohei Sato, Chiari Kojima, Tatsuo Shimosawa

**Affiliations:** a Department of Clinical Laboratory, International University of Health and Welfare Narita Hospital, Chiba, Japan; b Department of Clinical Laboratory, International University of Health and Welfare Mita Hospital, Tokyo, Japan

**Keywords:** Antibody, SARS-CoV-2, BNT-162b2 vaccine, Immunoassays, Immunological response

## Abstract

SARS-CoV-2-specific antibody measurement is important for evaluating COVID-19 vaccine efficacy. We quantified and compared anti-spike (S) antibodies using different commercial immunoassays. We tested serum samples from 70 SARS-CoV-2-naive health care workers 2 weeks after vaccination with a single dose of BNT162b2, 2 and 4 weeks, and 3 months after the second dose of BNT162b2. The following quantitative assays were used: Roche Elecsys *Anti*-SARS-CoV-2 S (Roche-S), Abbott SARS-CoV-2 IgG II Quant [Abbott-IgG(S)], and Abbott SARS-CoV-2 IgM (Abbott-IgM). All samples tested positive for Roche-S and Abbott-IgG antibodies after the second dose, with 83.6% Abbott-IgM positive rate. Roche-S and Abbott-IgG(S) correlated significantly in all samples (r = 0.920, p < 0.0001), and the Roche-S and Abbott-IgG(S) assay showed a strong correlation with each other at each time point after vaccination. Roche-S and Abbott-IgG(S) antibody titers were correlated with age; their rate of decline was age-dependent in males but not in females. Abbott-IgG(S) antibody titers decreased from 2 weeks after the second dose. Roche-S antibody titers peaked 2 weeks after the second dose in 76.2% of the participants; the titers recovered 3 months post-vaccination after declining at week 4 in 40.7% of the participants. The concordance between Roche-S and Abbott-IgG(S) antibody titers over time was 47.5%. Most participants presented significantly high Roche-S and Abbott-IgG(S) antibody titers after immunization. Some measurements were inconsistent with titer changes between these assays, possibly because of differences in the immunoglobulin-specificity of the kits.

## Highlights

• We compared *anti*-SARS-CoV-2 titers after BNT162b2 vaccination with immunoassay kits.
• Roche Elecsys and Abbott-IgG kits showed high sensitivity for antibody detection.
• Time-dependent changes in titers differed between Abbott-IgG and Roche assays.
• The specificity of immunoglobulin classes detected may explain these differences.

## 1 Introduction

Coronavirus disease 2019 (COVID-19) caused by severe acute respiratory syndrome coronavirus 2 (SARS-CoV-2) has become a pandemic [1]. In addition to treatment, prevention of the spread of COVID-19 is a necessary measure to overcome the pandemic, and vaccines against SARS-CoV-2 hold great promise for preventing COVID-19 spread [2,3]. In this context, the BioNTech/Pfizer BNT162b2 messenger RNA (mRNA) vaccine, the first COVID-19 vaccine to receive approval, shows efficiency and is being widely used [4].

SARS-CoV-2 elicits an immune response in the host with simultaneous production of IgG, IgA, and IgM antibodies [[5], [6], [7]]. A wide range of SARS-CoV-2 binding antibody assays has been developed based on different antigen targets and assay formats. The presence of nucleocapsid (N) protein antibodies indicates previous SARS-CoV-2 infection [6]. As the BNT162b2 vaccine supplies mRNA encoding only the spike (S) protein, only IgG, IgA, or IgM [S-receptor-binding domain (RBD)] antibodies against the S protein, and no antibodies against the N protein, are produced [[8], [9], [10]]. Therefore, the quantitative measurement of *anti*-SARS-CoV-2 S protein-specific antibodies may reflect the immune responses to vaccines or previous SARS-CoV-2 infection. Recent studies have shown that several serological assays, such as the Elecsys *Anti*-SARS-CoV-2 assay (Roche-S), Abbott Architect SARS-CoV-2 IgG II assay [Abbott-IgG(S)], and Abbott Architect SARS-CoV-2 IgM assay (Abbott-IgM) can reveal the dynamics of the humoral immune response in vaccinated individuals [[10], [11], [12], [13]]. The Roche kit quantitatively measures total immunoglobulin against the S protein, whereas the Abbott kit measures IgG and IgM separately. Neutralizing antibodies correlate with Roche-S or Abbott-IgG(S) antibodies [10,[14], [15], [16]] suggesting that the Roche-S or Abbott-IgG(S) antibody measurement may be a reliable indicator of vaccine efficacy. Overall, knowledge of the antibody response will contribute to our understanding of the immune response and appropriate prevention of COVID-19.

Several studies have examined antibody titers after vaccination using commercial antibody kits, showing valuable results. However, few studies have investigated the specific changes in antibody titers among vaccinated individuals. This study aimed to compare the titers using the Roche-S, Abbot-IgG(S), and Abbot-IgM kits in serum samples obtained from vaccinated healthcare workers without previous SARS-CoV-2 infection up to 3 months after the second dose.

## 2 Materials and methods

### 2.1 Study population and sample collection

A total of 70 healthcare workers from our affiliated hospitals, International University of Health and Welfare Narita Hospital (Chiba, Japan) and Mita Hospital (Tokyo, Japan), were included in this study. From March 2021 to May 2021, all study participants were vaccinated with the BNT162b2 from Pfizer-BioNTech. *Anti*-SARS-CoV-2 antibody measurements were performed before the first vaccine dose, 2 weeks after the first dose, and 2 weeks, 4 weeks, and 3 months after the second dose. All serum samples were aliquoted and stored at −80 °C until analysis.

### 2.2 Measurement using five different SARS-CoV-2 binding immunoassays

The characteristics of each assay are listed in Supplementary Table S1. The Roche and Abbott's assays were performed at Narita Hospital and Mita Hospital, respectively, in a double-blinded fashion. All samples were assessed using different assays according to the respective manufacturer's instructions. When measuring samples, calibration was performed using the controls recommended by the manufactures. To reduce errors in measurement, samples collected at each time point were assayed on the same later date.

### 2.3 Statistical analysis

Data analysis was performed using GraphPad Prism (Version 9.2.0., San Diego, CA, USA). A Kruskal-Wallis test was used for more than three groups and a Mann-Whitney *U* test for experiments with only two groups to analyze continuous variables such as antibody levels that did not meet normality assumptions. Spearman's correlation coefficients were calculated for correlations between the SARS-CoV-2 binding antibody assays. Linear regression was used to explore the association between age and antibody titers. A value of p < 0.05 was considered statistically significant.

### 2.4 Ethical statement

This study was approved by the institutional review board of our institutions (20-Nr-108). All participants included in the study signed an informed consent form, and the study complied with the Declaration of Helsinki of the World Medical Association.

## 3 Results

### 3.1 Characteristics of the study participants

This study involved 70 healthcare workers aged 22–64 years old, with a median age of 26.5 years. The participants were all Asian, and the group consisted of 48 female subjects and 22 male subjects (Supplementary Table S2). Serological tests were performed on the day of the vaccination, 2 weeks after the first dose, and 2 weeks, 4 weeks, and 3 months after the second dose. For tests in the 3-month category, blood samples were collected between 91 and 112 days after the second dose. Serum samples were tested using five *anti*-SARS-CoV-2 antibody assays (Supplementary Table S1), including N-protein-based immunoassays, Elecsys *Anti*-SARS-CoV-2 antibody (Roche-N) assay and Abbott SARS-CoV-2 IgG [Abbott-IgG(N)] assay. The results for both anti-nucleocapsid and anti-spike antibodies were negative for all participants on the day of vaccination.

### 3.2 Dynamics of changes in SARS-CoV-2 antibody levels after vaccination

**Fig. 1:**
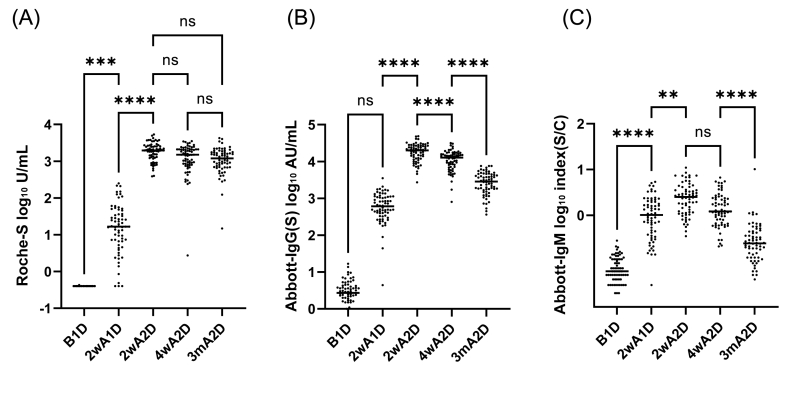
Dynamics of SARS-CoV-2-specific antibody titers in vaccinated individuals. SARS-CoV-2-specific antibody titers before vaccination (B1D, n = 70); at 2 weeks after administration of the first dose (2wA1D, n = 67); and at 2 weeks (2wA2D, n = 67), 4 weeks (4wA2D, n = 67), and 3 months (3mA2D, n = 66) after administration of the second dose. Y-axis represents logarithmic antibody levels of the Roche-S (A), Abbott-IgG(S) (B), and Abbott-IgM (C). The *horizontal line represents* the *median* of all values. The statistical significance of differences between groups was evaluated using the Kruskal–Wallis test in the GraphPad Prism software. **, p ≤ 0.01, ***, p ≤ 0.001, ****, p ≤ 0.0001.

Fig. 1 shows the dynamic changes in SARS-CoV-2 antibody levels during the observation period. The median antibody levels increased after the first dose determined by Roche-S (up to 16.6 U/mL) and Abbott-IgG(S) (up to 609.3 AU/mL) kits. Furthermore, the median antibody levels using Roche-S and Abbott-IgG(S) kits increased dramatically 2 weeks after the second dose [1973.0 U/mL for Roche and 20044.4 AU/mL for Abbott-IgG(S)]. The median Abbott-IgM response after the first dose was 1.0 index (S/C); after the second dose, the median response increased to 2.5 index (S/C). According to Abbott-IgG(S) and Abbot-IgM kits, antibody levels peaked 2 weeks after the second dose and then declined. According to Roche-S kit, levels showed a similar peak after 2 weeks and remained high for 3 months after the second dose. For N-protein-based immune-assays (Abbott SARS-CoV-2 IgG and Elecsys *Anti*-SARS-CoV-2 antibody), titers remained unchanged after vaccination (Supplementary Table S3).

### 3.3 Assessment of the positivity rate

We assessed the positivity rates of the sera samples using the antibody assays based on the manufacturer's recommended cut-off values (Supplementary Table S1). The positivity rates of Roche-S and Abbott-IgG(S) assay after the first dose were 94.0% (63/67) and 97.0% (65/67), respectively. After the second dose, all samples were positive and remained during the study period in both the Roche-S and Abbott-IgG(S) assays. The positivity rate for Abbott-IgM peaked at 2 weeks after the second dose and declined to 7.6% (5/66) when measured 3 months after the second dose (Supplementary Table S4).

### 3.4 Comparison of serum chemiluminescence immunoassay methods

**Fig. 2:**
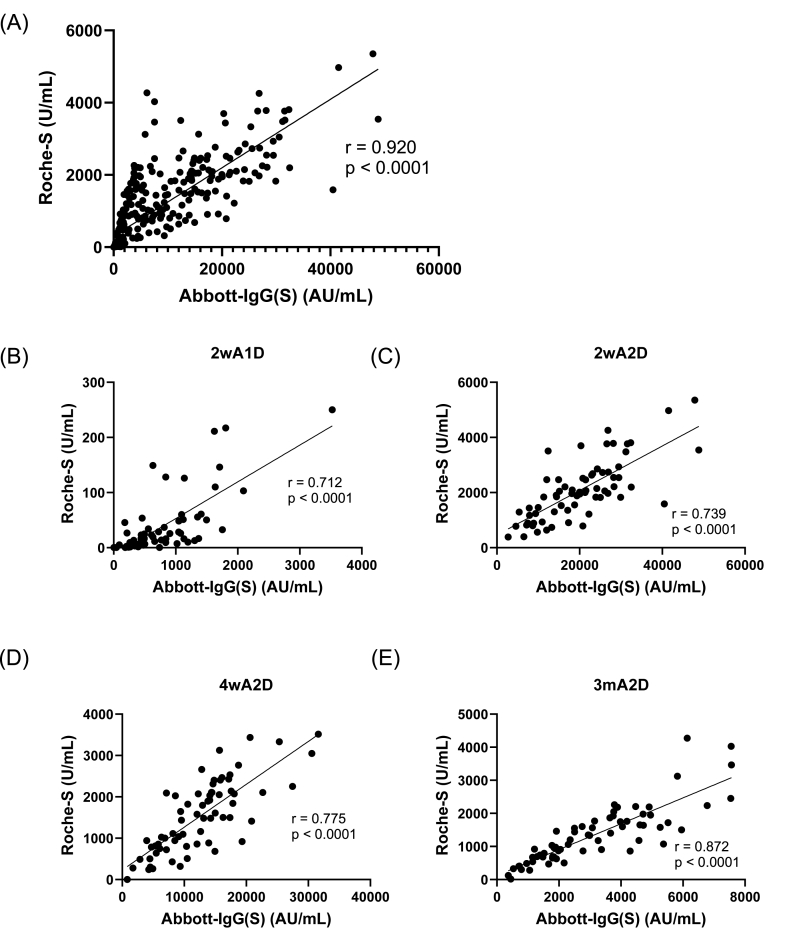
Correlations between each pair of tests for Roche-S and Abbott-IgG(S). Correlation of Roche-S with Abbott-IgG(S) antibody titers is shown for (A) all samples (n = 336); (B) 2 weeks after administration of the first dose (n = 67); and (C) 2 weeks (n = 67), (D) 4 weeks (n = 67), and (E) 3 months after administration of the second dose (n = 66).

In this study, 336 serum samples were collected from 70 participants before and after vaccination. As shown in Fig. 2A, a strong correlation (r = 0.920, p < 0.0001) was observed between the antibody levels in all samples, according to the Roche-S and Abbott-IgG(S) kits. Using Spearman correlation coefficients, the titer levels of Roche-S, Abbott-IgG(S), and Abbott-IgM were analyzed from the date of the first dose to 3 months after the second dose (Table 1). Antibody levels based on the Roche-S and Abbott-IgG(S) assay showed a strong correlation with each other at each time point after vaccination (Fig. 2). There were also weak but significant correlations between antibody levels resultant from Abbott-IgG(S) and Abbott-IgM and between Roche-S and Abbott-IgM assays, but no correlations were observed at 2 weeks after the second dose for Roche-S (Table 1).

**Table 1:** Correlations between antibody levels for Roche-S, Abbott-IgG(S), and Abbott-IgM at different time points after vaccination.

		After 1st dose		After 2nd dose					
		2 weeks		2 weeks		4 weeks		3 months	
		**Abbott-IgG(S)**	**Abbott-IgM**	**Abbott-IgG(S)**	**Abbott-IgM**	**Abbott-IgG(S)**	**Abbott-IgM**	**Abbott-IgG(S)**	**Abbott-IgM**
**Roche-S**	**r**	0.712	0.571	0.739	0.177	0.775	0.275	0.872	0.385
	**p value**	<0.0001	<0.0001	<0.0001	ns	<0.0001	0.0241	<0.0001	0.0014
**Abbott-IgG(S)**	**r**		0.662		0.327		0.374		0.361
	**p value**		<0.0001		0.007		0.0018		0.0029

### 3.5 Differences in dynamics of *anti*-SARS-CoV-2 antibody levels after vaccination

Serum samples from 21 male and 48 female participants, with a median age of 26.0 years (IQR: 24.0–37.5), were analyzed during the complete course of the study. In addition, we investigated the antibody response in each participant.

**Fig. 3:**
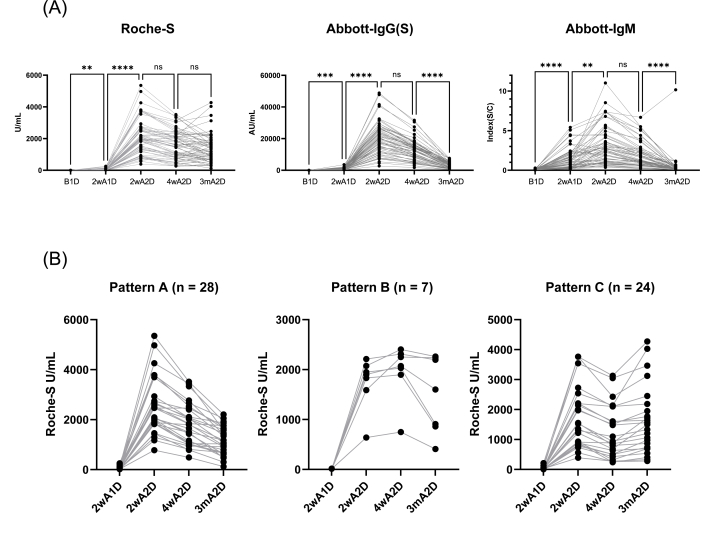
Dynamics of SARS-CoV-2-specific antibody titers after vaccination. (A) Changes in Roche-S, Abbott-IgG(S), and Abbott-IgM titers in individuals after vaccination. (B) Three different patterns of the Roche-S antibody response. Pattern A: Antibody titers peaked 2 weeks after the second administration and then declined. Pattern B: Antibody titers peaked 4 weeks after the second administration and then declined. Pattern C: Antibody titers decreased 4 weeks after the second administration and then increased again.

In the Abbott-IgG(S) assay, all participants exhibited peak antibody levels after 2 weeks of the second dose, followed by a decline at 4 weeks (median 37.4%) and a subsequent decline at 3 months (median 84.1%) (Fig. 3A). According to the Abbott-IgM assay, in all but two cases, antibody levels peaked at 2 weeks after the first dose or 2 weeks after the second dose and then decreased. One participant had the highest antibody levels by Abbott-IgM at 3 months after the second dose and was negative for COVID-19. The Roche-S assay showed that antibody levels peaked 2 weeks after the second dose in 76.2% (45/59) of the participants, and 4 weeks and 3 months after the second dose in 11.9% (7/59) and 11.9% (7/59) of the participants, respectively (Fig. 3A).

Interestingly, according to the Roche-S kit, antibody levels displayed three different patterns over time (Fig. 3B). Patterns A and B showed increased antibody levels in the second or fourth week after the second dose. For Pattern C, a decline in antibody from 2 weeks to 4 weeks and another increase at 3 months were observed. Among these three patterns, we did not find any differences in age, sex, or IgG and IgM class of antibody levels measured by the Abbott kit (data not shown). However, there was a tendency for a greater rebound of Roche antibody levels in younger age groups (Pattern A, 33.4 ± 13.1 years: Pattern B, 36.7 ± 11.8 years: C, 29.5 ± 9.3 years).

### 3.6 Simple linear regression of age or sex versus antibody levels

**Fig. 4:**
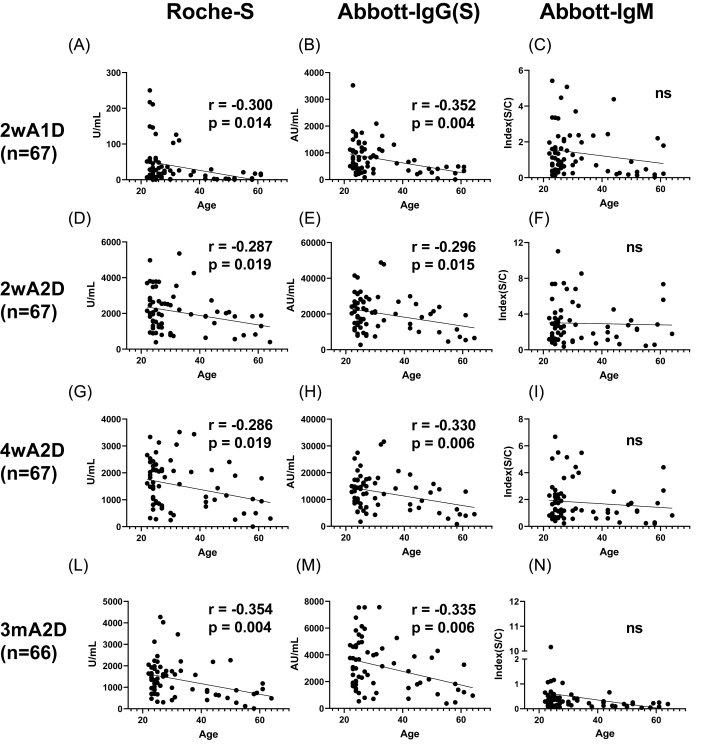
Scatter plot of the participants' ages by Roche-S (A, D, G, L), Abbott-IgG(S) (B, E, H, M), or Abbott-IgM (C, F, I, N) antibody titer at different time points after vaccination. A–C data refer to 2 weeks after the first dose (2wA1D, n = 67). D–F data refer to 2 weeks after the second dose (2wA2D, n = 67). G–I data refer to 4 weeks after the second dose (4wA2D, n = 67). L–N data refer to 3 months after the second dose (3mA2D, n = 66). The horizontal line represents the median of all values.

**Fig. 5:**
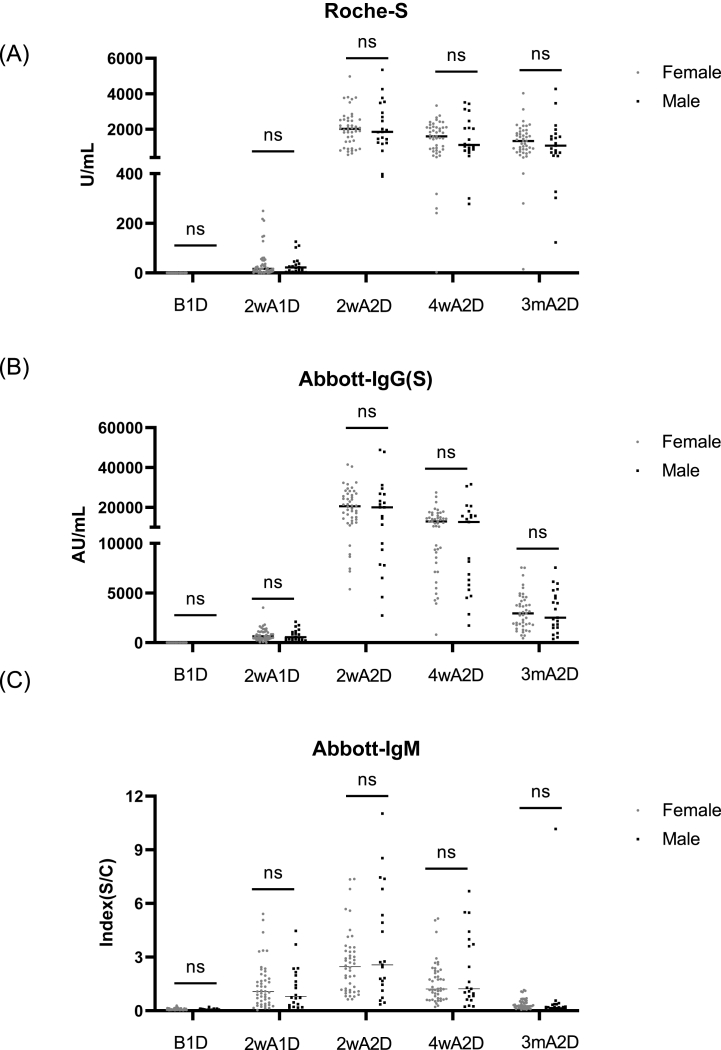
Distribution of antibody levels in females (gray dots) and males (black dots). Titers of antibodies measured by Roche-S (A), Abbott-IgG(S) (B), and Abbott-IgM (C) at baseline, 2 weeks after the first dose, 2 weeks after the second dose, 4 weeks after the second dose, and 3 months after the second dose.

The potential effects of age or sex on antibody levels at each time point after the first and second dose were examined. Age-dependent negative declines were found for the Roche-S and Abbott-IgG(S)-based levels using simple linear regression (Fig. 4). In contrast, we could not find any correlation between Abbott-IgM levels and age. Moreover, no differences were found in Roche-S or Abbott-IgG(S) levels between males and females at any time point after vaccination (Fig. 5).

### 3.7 Age- or sex-related differences in the rate of antibody decrease after vaccination

**Fig. 6:**
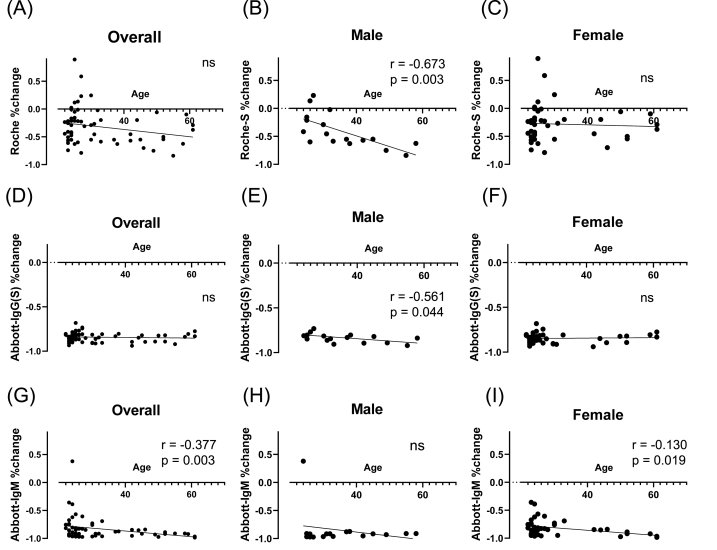
Comparison of the percentage change in antibody levels from peak to 3 months after the second dose. Rate of change in Roche-S (A–C), Abbott-IgG (S) (D–F), and Abbott-IgM antibody titers (G–I) according to age for all subjects (A, D, G), 17 males (B, E, H) and 42 females (C, F, I) are shown. Lines indicate simple linear regression.

The correlation between age and percentage decline in antibody levels from the peak observed 3 months after the second dose was examined in serum samples collected from 17 male and 42 female participants with a median age of 26.0 years (IQR: 24.0–38.0) throughout the study. Females are younger (median age 25.0 years) than males (32.0 years, p < 0.05). Comparison of changes in antibody levels with age revealed no significant differences for Roche-S and Abbott-IgG(S) (Fig. 6A and D), but Abbott-IgM antibody levels did change with age (Fig. 6G). When age and rate of change in antibody levels were examined by sex, a correlation was found in males [r = −0.673, p = 0.003 for Roche-S; r = −0.561, p = 0.044 for Abbott-IgG(S)] (Fig. 6B and E) but not in females (Fig. 6C and F). Conversely, the rate of change of Abbott-IgM antibody levels correlated with age only in females (r = −0.130, p = 0.019) (Fig. 6I).

## 4 Discussion

This study shows that the Roche-S, Abbott-IgG(S) and Abbott-IgM assays are sensitive and perform well for vaccinated healthcare workers. In general, peak antibody levels were observed 2 weeks after the second dose, and they declined 3 months after the second dose. Recent studies have shown a correlation between antibody levels based on Abbott and Roche kits in vaccinated individuals [10,13,14] as well as in patients with COVID-19 [17]. In the present study, the Roche-S and Abbott-IgG(S) assay results correlated highly for all serum samples collected before and after vaccination. Furthermore, the Roche-S assay showed a strong correlation with the Abbott-IgG(S) assay at all time points. Nevertheless, antibody levels by Roche-S correlated with those by Abbott-IgM only 2 weeks after the first dose and 4 weeks after the second dose; there was a weak correlation between Abbott-IgM and Abbott-IgG(S) up to 4 weeks after the second dose.

Consistent with a prior study [12], Abbot-IgM antibody levels showed a high (83.6%) positivity rate 2 weeks after the second dose. At 3 months after the second dose, the Abbott-IgM antibody levels declined and were positive in only 7.6% of cases. Abbott-IgM antibody levels in COVID-19 patients reportedly increase during the first 3 weeks after the onset of symptoms, decrease during the first 4–5 weeks, and remain positive in most patients during the first 2 months [18]. In our study, Abbott-IgM antibody levels of the vaccinees were low, and the positivity rate decreased quickly 4 weeks after the second dose. In addition, some vaccinees were negative for Abbott-IgM antibody levels. The dynamics of IgM antibodies in SARS-CoV-2 [7,12] and other infectious diseases are well known. The fact that IgM levels decline several weeks after infection or vaccination [12,19] and that IgM levels measured several weeks after vaccination are of limited value is acceptable.

Differences related to sex in the production of *anti*-SARS-CoV-2 antibodies remain controversial. Some studies have reported that males show lower *anti*-SARS-CoV-2 antibody titers [11,20] than females do, whereas some studies have found no sex differences [[21], [22], [23]]. In our study, although more samples were obtained from young females, there were no sex differences in antibody levels based on all three tests over the entire period of this study (Fig. 5).

It has been reported that antibody levels measured with Roche-S [11,20,24] and Abbott-IgG(S) [9,19,23] after vaccination show an age-dependent decrease. In our study, we also found differences in antibody levels according to age for the Roche-S assay at each time point of blood collection (Fig. 4). Similarly, Abbott-IgG(S) antibody levels correlated negatively with age. However, no correlation with age was found for Abbott-IgM antibody levels.

Furthermore, we investigated the relationship between age and relative change in antibody levels at 3 months after the second dose against the peak titer in 59 patients collected over the whole period. Overall, the decline antibody titer correlated with age (Fig. 4), however age did not affect the rate of decline in antibody titer measured by the Roche-S assay kit or Abbott-IgG(S) kit (Fig. 6). Of note, there were sex-related differences in the rate of antibody decline in the age correlation. As depicted in Fig. 6, the age-dependent decline in antibody titer was only apparent in males. Several possible mechanisms of sex-related differences in antibody production have been reported: estrogen-induced promotion of somatic hypermutation [25], reduced stringency in selection against autoreactive B cells [26,27], sex differences in germinal center formation [28] and epigenetic accessibility of B cell loci [29]. There is increasing evidence that COVID-19 causes more severe symptoms and higher mortality among males than females [30]. Sex differences also influence variances in immune responses to COVID-19 [31,32]. Sex-related differences in immunity also have implications for vaccine responses. Supporting this hypothesis, females exhibit greater antibody responses and more adverse events after vaccination than do males [33,34]. Hormonal and genetic dissimilarities are likely involved in the mechanism of sex-related differences in vaccine-induced immunity [35]. Although we are unable to speculate possible mechanisms based on data from the current study, the results of the changes in antibody levels suggest sex-related differences in responsiveness to the BNT162b2 vaccine. The effect of age differences should be considered because the ages of male and female participants were different.

Roche-S and Abbott-IgG(S) assays focus on detecting antibodies against the RBD of the spike (S) protein. Although a good correlation was found between Roche-S and Abbott-IgG(S), the classes of immunoglobulin assessed differ between these kits. The Abbott assay specifically measures IgG or IgM against the S protein; the Roche assay kit mainly detects IgG, IgM, and IgA classes against the S protein. Roche-S antibody levels increase after infection or vaccination and then decrease, but some studies have shown that absolute Roche-S antibody levels vary among individuals [11,36,37]. In this study, the Abbott kit showed a significant reduction in antibody titers 3 months after the second dose, whereas the Roche-S kit showed no significant reduction in antibody titers (Fig. 1, Fig. 3). Interestingly, as illustrated in Fig. 3B, the Roche-S assay showed different time-dependent patterns in antibody levels after the second dose. Several studies have suggested that IgA antibody levels increase in the later phase after vaccination [5,38], and it has been reported that, in the phase when levels of IgM and IgG antibodies against spike decrease, IgA antibodies are barely affected [5]. The Roche-S kit detects all immunoglobulin classes including IgA antibodies against SARS-CoV-2 in addition to IgG and IgM classes. In our study, Roche-S antibodies decreased and then rebounded in 40.7% (24/59) of subjects (Fig. 3B). Despite no statistically significant differences related to age or sex in the variations in Roche-S antibody levels, the rebound in Roche-S antibody levels was more likely to be seen in younger age groups. This study was performed with healthy and rather young participants, and to generalize this finding, further studies with a wide variety of age groups is required. Indeed, our result cannot be explained by differences in underlying diseases; it is possible that IgA antibody titers may increase during the observational period and affect the antibody titers monitored by the Roche-S kit. We did not directly measure IgA class against SARS-CoV-2, and further research is required.

All samples were negative for Abbott-IgG(N) antibodies during the study period (Supplementary Table S3). Since the BNT162b2 vaccine provides mRNA encoding only the spike(S) protein, vaccination did not result in an increase in Abbott-IgG(N) antibodies and the negative result implies that SARS-CoV-2 infection did not occur in our subjects.

This study has several limitations, including the small sample size, a large proportion in young-aged groups, especially females, and a small proportion of males. We included subjects without SARS-CoV-2 infection, and all samples were negative for Abbott-IgG(N) during the study period. However, it should be noted that Abbott-IgG(N)-negative results alone do not completely rule out the possibility of SARS-CoV-2 infection. In addition, certain risk groups were not included, such as elderly patients and patients with serious complications. Other factors influencing antibody levels were not fully investigated, except for age and sex. Moreover, this study focused only on the BNT162b2 vaccine, as it was the first vaccine to be licensed and administered to healthcare workers in Japan. Additionally, the short survey period was a limitation considering that the study aimed to evaluate medium-to long-term changes in antibodies. The assays used in this study did not directly measure the levels of neutralizing antibodies; therefore, the results cannot be used as a reference for the effectiveness of infection prevention.

Overall, our data show that following the BNT162b2 mRNA vaccination, the levels of SARS-CoV-2 antibodies eventually increase. High antibody titers in the Roche-S and Abbott-IgG(S) kits were maintained after vaccination, whereas Abbott-IgM showed a lower positive rate at 3 months after the second dose. After the second dose, the time-dependent pattern of antibody levels differed between the Abbott-IgG(S) and Roche-S assays. This discrepancy could be attributed to the different antibody classes being measured. We must recognize the differences in specificity of antibody classes among the kits. Furthermore, the combination of Roche-N, Roche-S, Abbott-IgG(N), and Abbott-IgG(S) shows the benefits of serological tests when distinguishing between a prior infectious state and a vaccine response. Further data is needed to investigate long-term changes in antibody levels measured by the Roche and Abbott kits after vaccination and the causes of the differences in antibody titer variation.

## Ethics statement

This study was initiated after approval from the Ethical Review Board of the International University of Health and Welfare (approval number 20-Nr-108).

## Author contribution statement

Miku Nakai; Daisuke Yokoyama; Tomoaki Sato; Ryohei Sato: Performed the experiments.

Chiari Kojima: Analyzed and interpreted the data; Wrote the paper.

Tatsuo Shimosawa, MD, PhD: Conceived and designed the experiments; Analyzed and interpreted the data; Contributed reagents, materials, analysis tools or data; Wrote the paper.

## Data availability statement

Data included in article/supp. material/referenced in article.

## Declaration of competing interest

The authors declare that they have no known competing financial interests or personal relationships that could have appeared to influence the work reported in this paper.
