# Supplementary material for: Variation in antibody titers determined by Abbott and Roche Elecsys SARS-CoV-2 assays in vaccinated healthcare workers
Source: Heliyon. 2023 May 22;9(6):e16547. doi: 10.1016/j.heliyon.2023.e16547 (PMC10201891; doi:10.1016/j.heliyon.2023.e16547)
Supplement: Multimedia component 1 [file mmc1.docx]

## **Supplementary Table S1. Characteristics of the five SARS-CoV-2 antibody assays**

|  | **Roche** | | **Abbott** | | |
| --- | --- | --- | --- | --- | --- |
|  | Roche Diagnostic, Basel, Switzerland | | Abbott, Chicago, IL, USA | | |
| Product Name | Elecsys Anti-SARS-CoV-2 | Elecsys Anti-SARS-CoV-2 S | Architect SARS-CoV-2 IgG | Architect SARS-CoV-2 IgG II Quant | Architect SARS-CoV-2 IgM |
| Analyzer | Elecsys Cobas e801 | | Architect | | |
| Principle | ECLIA | ECLIA | CMIA | CMIA | CMIA |
| Antigen Target | Nucleocapsid, total | Anti-RBD, total | Nucleocapsid, IgG | Anti-RBD, IgG | Anti-RBD, IgM |
| Cut-off value | 1.0 U/mL | 0.8 U/mL | 1.4 Index (S/C) | 50 AU/mL | 1.0 Index (S/C) |

ECLIA, electrochemiluminescence immunoassay; CMIA, chemiluminescence microparticle immunoassay;　　　　RBD, receptor-binding domain; AU, arbitrary unit; S/C, signal-to-cutoff ratios

## **Supplementary Table S2. Characteristics of the study participants**

| Sex |  |  |  |  |
| --- | --- | --- | --- | --- |
| Male (%) | 22 (31.4) | | |  |
| Female (%) | 48 (68.6) | | |  |
| Age, median (Inter Quartile Range) | 26.5 (24-37.8) | | |  |
| Prevaccination antibody levels |  |  |  | Cut-off value |
| Abbott IgG(N) index (S/C) | 0.058 | ± | 0.158 | < 1.4 |
| Abbott IgG(S) (AU/ml) | 3.448 | ± | 3.020 | < 50 |
| Abbott IgM index (S/C) | 0.080 | ± | 0.052 | < 1.0 |
| Roche Anti-SARS-CoV (U/mL) | 0.098 | ± | 0.092 | < 1.0 |
| Roche Anti-SARS-CoV-2 S (U/mL) | 0.400 | ± | 0.003 | < 0.8 |

## **Supplementary Table S3.** Antibody levels measured by the N-protein-based immuno-assays before and after vaccination

|  | **Before vaccination** | | | **1st dose** | | | **2nd dose** | | | | | | | | |
| --- | --- | --- | --- | --- | --- | --- | --- | --- | --- | --- | --- | --- | --- | --- | --- |
|  |  |  |  | **2 weeks** | | | **2 weeks** | | | **4 weeks** | | | **3 months** | | |
| **Roche-N U/mL** | 0.098 | ± | 0.092 | 0.087 | ± | 0.008 | 0.118 | ± | 0.139 | 0.090 | ± | 0.008 | 0.092 | ± | 0.007 |
| **Abbott-IgG(N)**  **index (S/C)** | 0.058 | ± | 0.158 | 0.059 | ± | 0.149 | 0.072 | ± | 0.155 | 0.079 | ± | 0.199 | 0.049 | ± | 0.077 |

## **Supplementary Table S4.** Positive rate of Roche-S, Abbott-IgG(S), and Abbott-IgM assays

|  | **After 1st dose** | **After 2nd dose** | | |
| --- | --- | --- | --- | --- |
|  | **2 weeks** | **2 weeks** | **4 weeks** | **3 months** |
| **Roche-S** | 0.940 | 1.000 | 1.000 | 1.000 |
|  | (63/67) | (67/67) | (67/67) | (66/66) |
| **Abbott-IgG(S)** | 0.970 | 1.000 | 1.000 | 1.000 |
|  | (65/67) | (67/67) | (67/67) | (66/66) |
| **Abbott-IgM** | 0.507 | 0.836 | 0.642 | 0.076 |
|  | (34/67) | (56/67) | (43/67) | (5/66) |
